# Mechanisms of change and heterogeneous treatment effects in psychodynamic and cognitive behavioural therapy for patients with depressive disorder: a randomized controlled trial

**DOI:** 10.1186/s40359-021-00517-6

**Published:** 2021-01-22

**Authors:** J. I. Røssberg, J. Evensen, T. Dammen, T. Wilberg, O. Klungsøyr, M. Jones, E. Bøen, R. Egeland, R. Breivik, A. Løvgren, R. Ulberg

**Affiliations:** 1 Division of Mental Health and Addiction, Oslo University Hospital, P.O. Box 4959, 0424 Nydalen, Oslo, Norway; 2 Institute of Clinical Medicine, University of Oslo, P.O. Box 1171, 0318 Blindern, Oslo, Norway; 3 Nydalen Outpatient Clinic, P.O. Box 4959, 0424 Nydalen, Oslo, Norway; 4 Department of Behavioural Science in Medicine, Faculty of Medicine, University of Oslo, Oslo, Norway; 5 Department of Psychiatry, Diakonhjemmet Hospital, Oslo, Norway; 6 Division of Psychiatric Treatment Research, Oslo University Hospital, Oslo, Norway

**Keywords:** Major depressive disorder, Cognitive behavioral therapy, Psychodynamic therapy, Mediators, Moderators

## Abstract

**Background:**

Major depressive disorder (MDD) is a prevalent psychiatric condition associated with significant disability, mortality and economic burden. Cognitive behavioral therapy (CBT) and psychodynamic psychotherapy (PDT) are found to be equally effective for patients with depression. However, many patients do not respond sufficiently to either treatment. To offer individualized treatment, we need to know if some patients benefit more from one of the two therapies. At present little is known about what patient characteristics (moderators) may be associated with differential outcomes of CBT and PDT, and through what therapeutic processes and mechanisms (mediators) improvements occur in each therapy mode. Presently only theoretical assumptions, sparsely supported by research findings, describe what potentially moderates and mediates the treatment effects of CBT and PDT. The overall aim of this study is to examine theoretically derived putative moderators and mediators in CBT and PDT and strengthen the evidence base about for whom and how these treatments works in a representative sample of patients with MDD.

**Methods:**

One hundred patients with a diagnosis of MDD will be randomized to either CBT or PDT. Patients will be treated over 28 weeks with either CBT (one weekly session over 16 weeks and three monthly booster sessions) or PDT (one weekly session over 28 weeks). The patients will be evaluated at baseline, during the course of therapy, at the end of therapy, and at follow-up investigations 1 and 3 years post treatment. A large range of patient and observer rated questionnaires (specific preselected putative moderators and mediators) are included.

**Discussion:**

The clinical outcome of this study may better guide clinicians when deciding what kind of treatment any individual patient should be offered. Moreover, the study aims to further our knowledge of what mechanisms lead to symptom improvement and increased psychosocial functioning.

***Trial registration*:**

ClinicalTrials.gov Identifier: NCT03022071.

## Background

Major depressive disorder (MDD) is a prevalent psychiatric condition associated with significant disability, mortality and economic burden for society. MDD is the fourth leading cause of disease burden worldwide and is expected to rank first in high-income countries by the year 2030 [62, 86]. Given this tremendous disease burden, there is an urgent need for efficient and individualized treatments for the disorder. Psychotherapy and antidepressant medications constitute the predominant treatments for MDD [61] and large meta-analyses have concluded these to be equally effective [24, 25].

Cognitive Behavioral Therapy (CBT) and Psychodynamic Psychotherapy (PDT) are two of the most widely used and researched psychological treatments of depression. Large Meta analyses have shown PDT and CBT to be on average equally effective when treating depression, and to be clearly more effective than no treatment [25, 76]. Although psychotherapy is an effective treatment, Driessen et al. found that a greater portion of patients do not respond sufficiently [28], furthermore relapse rates of up to 40% are reported [75]. “What works for whom and how?” is currently one of the main challenges in psychotherapy research [53]. There is, however, a critical lack of knowledge of what treatment is most beneficial for the individual patient, and valuable time is wasted when patients don’t first hand receive the treatment that most benefit them [1].

Research projects in this field tend to be few, and suffer from significant methodological shortcomings. The majority of previous process-outcome studies have been correlational studies, and not randomized controlled trials, most without assessments during treatment, making research on mechanisms and causal inference difficult. There is further a lack of clear findings, and results have been difficult to replicate.

## What works for whom? (Moderators)

Patients with MDD differ on several clinical aspects such as levels of symptom severity, comorbidity and psychosocial functioning. The empirical base is, however, scarce regarding which clinical characteristics are associated with differential efficacy in PDT and CBT for depression [27].

A well powered RCT by Driessen et al. [27] found similar efficacy for CBT and PDT. When comparing subgroups of patients, CBT was found to be more advantageous in patients with comorbid anxiety and PDT more advantageous in patients with a longer duration of current depressive episode **(**more than one year). The authors hypothesize that patients with longer duration of depressive symptoms are more likely to have a vulnerable personality structure, obtain a less optimal working alliance, and experience more negative transference feelings towards their therapist. PDT therapists could be better trained to approach these relational aspects during therapy. This study did not, however, examine the included patients with regard to personality disorders (PD) or traits.

A review by Friborg and colleagues found that 50% of patients with depression had one or more co-occurring PD diagnoses [31]. It has been suggested that long-term PDT might be better suited for patients with complex mental disorders, such as PDs [55]. Other studies propose that CBT better suits patients with personality traits such as low emotional awareness or a distancing attachment style [5, 63, 68].

Some studies have compared CBT to Interpersonal therapy (IPT), which is relatively closer to PDT than CBT as it focuses on patient’s relational difficulties. A study from 2011 that randomized depressed patients to CBT and IPT found that patients with more comorbid PD diagnoses responded better to CBT than to IPT [21]. A comparable RCT from 2007 found that patients with avoidant PD or traits benefitted more from CBT than IPT [49].

Four studies [8, 16, 49, 63] have examined personality traits or disorders as moderators of response to IPT and CBT. Overall, the findings suggest that both borderline PD and an avoidant attachment style predict better response to CBT, while the results of one of the studies suggest that patients with obsessive–compulsive PD may experience better response to IPT [8]. These studies were limited by low number of participants. Other studies have found the presence of PD to predict poorer outcome across treatments when comparing CBT to IPT [44]. Thus, whether a co-occurring PD or a distancing attachment style moderate outcome of CBT versus short-term PDT is not known.

The therapeutic alliance refers to the relationship between the therapist and the patient, and to what extent they agree on treatment goals and therapeutic tasks, and form a positively toned emotional bond 2013,55. The alliance is considered a common factor in psychotherapy (e.g. therapeutic alliance, patient expectancy, therapist competence and adherence to the therapy model), and has been shown through meta-analyses to influence outcome equally across different variants of psychotherapy [42]. These meta-analyses have, however, examined the influence of alliance on a group level rather than as a moderator of treatment in subgroups of patients. Few previous studies have explored how alliance may moderate outcomes across various treatments, and even fewer studies have focused on interactions with patient characteristics as possible moderators [59]. Consequently, we still do not know whether alliance is more important in certain therapies, or for certain patients [6].

Adult attachment style has been identified as a patient variable that potentially influence alliance. Attachment theory, originally articulated by Bowlby [19], proposes that individuals’ representations of interpersonal relationships stem from early experiences with caretakers, and that particular patterns of relating, called adult attachment styles, develop as a result [30, 57, 64]. Levy et al. [58] conducted a meta‐analysis of 36 studies (3158 patients) which suggested that patients with secure attachment style pretreatment attained better psychotherapy outcome compared to insecurely attached patients. It was also found that greater improvement in attachment security predicted greater improvement in outcome. However, baseline attachment did not predict dropout. Preliminary moderator analyses suggested that those who experience low pretreatment attachment security may achieve better treatment outcome in therapy that include a focus on interpersonal interactions and close relationships.

The Quality of Object Relations (QOR) is a measure of difficulties in relational functioning and the quality of previous and current relationships. QOR has been shown to be a predictor and moderator of treatment response in PDT [40]. However, to what extent QOR acts as a moderator in CBT has not been explored.

## How can we better select treatment based on patient characteristics?

Previous research has mainly examined moderators as single units. Could we see a stronger moderator effect by examining clusters of patient characteristics that moderates treatment outcome in similar direction? More recently DeRubeis et al. [26] used data from a trial of antidepressant medications versus CBT for MDD to calculate predictions of post-treatment scores on the Hamilton Rating Scale for Depression (HRSD) in each of the two treatments. Five pre-randomization variables that predicted differential response (marital status, employment status, life events, comorbid PD, and prior medication trials) were included in regression models, permitting the calculation of each patient’s Personalized Advantage Index (PAI). The study reported that for 60% of the sample a clinically meaningful advantage was predicted for one of the treatments, relative to the other. When these patients were divided into those randomly assigned to their ‘‘Optimal’’ treatment versus those assigned to their ‘‘Non-optimal’’ treatment, outcomes in the former group were superior. In another study the PAI were used to optimize treatment outcome in CBT and IPT [44]. The study found six moderators (somatic complaints, cognitive problems, paranoid symptoms, interpersonal self-sacrificing, attributional style and number of life events). As much as 63% of the patients had a clinical advantage in either CBT or IPT**.** In a recently published study, Cohen et al. [22] describes how use of four different statistical techniques can improve the ability to identify the best possible treatment for patients with a depressive disorder. They showed that patients with the strongest PAIs, had an effects size of 0.37 for receiving the indicated versus contraindicated treatment. This method could prove to be valuable not only in providing better evidence for moderators of psychotherapy, but also give a better understanding of how moderators influence each other. Treatment selection based on clusters of moderators may significantly increase the chances of establishing the optimal treatment for the individual patient.

## How does psychotherapy work? (Mediators)

A mediator of treatment outcome is a specific mechanism of change for a particular form of psychotherapy, suggesting how or why the change in symptoms is obtained [9, 52]. Psychotherapy is a complex process, and there is probably not one single mechanism of change, but several factors working together. Still, to show that there is causality between changes in one mediator variable and outcome is methodologically challenging and would be an important step forward. Hence, the biggest challenge in this field is to demonstrate the causality between change in the mediator and reduction in depressive symptoms [56].

A central theoretical guideline in CBT is that changing different cognitive processes is followed by less symptoms and increase in functioning and quality of life. Regarding MDD, the cognitive model hypothesizes that the underlying cognitive schemas (depressogenic schemas) are changed through CBT [33]. Deactivating the depressogenic schemas may reduce depressive symptoms. The cognitive schemas include negative automatic thoughts, dysfunctional attitudes, different attributional styles and cognitive distortions, which could all be mediators of change in CBT. Furthermore, it is hypothesized that CBT works by teaching the patients different and more appropriate skills to handle their symptoms (compensatory skills) [12].

In PDT theoretically assumed mediators of change are improved self-understanding or insight, increased emotional awareness, a less harsh self-view, more mature defense mechanisms, and increased reflective functioning.

In psychodynamic theory it is assumed that improved self-understanding or insight may lead to successive improvements in symptoms [47]. An increased self-understanding is, according to theory, followed by better coping mechanisms to stress and an ability to choose adaptive interpersonal and health promoting behavior [55]. Furthermore, it is hypothesized that PDT will increase the patients’ capacity for reflective functioning [58] which consequently could lead to less depressive symptoms.

An attempt to uncover mechanisms of change in CBT was reported by Rush and colleagues already in 1981, when they examined 35 patients with depression who received CBT or an antidepressant. They found that changes in cognitive processes predicted significantly lower depression scores at follow up in the CBT group but not in the antidepressant group. They suggested that this could indicate an indirect support for the cognitive theory of depression [72]. This is in line with several other studies that have examined how cognitive processes might mediate outcome among patients with a depressive disorder. In a study by Kaufman and colleagues examining eight potential mediators, they found that changes in negative automatic thoughts were related to outcome in CBT for adolescents with depression. However, they had only pre and posttest assessments making inferences of causality difficult [50]. Tang and DeRubeis found that change in cognitions was followed by less depressive symptoms in the following sessions [77].

Furthermore, a number of studies have shown that CBT is associated with reduced negative automatic thoughts and that these changes co-vary with depressive symptomatology [7, 67, 87]. However, these aforementioned studies mostly did not examine if cognitive change happened before the changes in mood (temporality), or whether the changes were specific for CBT. It could be that negative automatic thoughts are a product of depression rather than a cause of depression.

As summarized in Bergin and Garfield’s Handbook of Psychotherapy and Behavior Change [53], theory of change in CBT is proposed as change in dysfunctional attitudes, underlying schemas and compensatory skills. However, no adequate test of these mediators has been done to date. Previous studies are limited by not including a control/comparison group, inadequate sample size, and furthermore fail to assess the mediator before the symptom change. Bergin and Garfield conclude that further studies should examine which variables mediate the effects of CBT and to what degree such variables are specific to CBT.

Studies of change mechanisms in PDT have focused on insight (self-understanding), reflective functioning and defense mechanisms. In a recently published systematic review including 22 studies, Jennissen and co-workers found a moderate correlation between insight/self-understanding and outcome [47]. However, most of the studies are correlational and further studies are needed to establish insight as a mediator. It has been concluded that insight might be a relevant mechanism of change across different treatment modalities [47]. However, further longitudinal research is necessary to provide evidence for insight as a putative mediator [47].

Reflective functioning (RF) or mentalizing is defined as the capacity to perceive human behavior as expressions of mental states, like thoughts, affects, dreams and intentions [29]. Very few studies have investigated RF as a potential mediator of clinical outcome, and the results are mixed. Levy et al. [58], included 90 patients with borderline PD and found significant changes in RF in PDT compared to dialectical behavior therapy. However, they did not explore changes in RF relative to symptoms. In a study of 44 patients with PDs given inpatient PDT the average level of RF did not increase and there was no association between change in RF and clinical improvement [83]. RF is also proposed as a common factor across various forms of psychotherapy [10], and as pointed out by Crits-Christoph et al. in Handbook of Psychotherapy and Behavior Change [53], RF needs to be studied further to examine whether it could be a putative mediator and specific for PDT.

Defense mechanisms are processes initiated unconsciously to avoid experiencing conflict or anxiety. Two studies have shown that exploration of defense mechanisms are an effective part of the therapeutic process in PDT. Johansen et al. found, among 40 patients with PDs, that changes in defensive functioning significantly predicted change in symptoms [42]. Bond and Perry [18] found that changes in overall defensive functioning were significantly associated with decrease in depressive symptoms among patients who received 3–5 years of psychoanalytic therapy. It will be important to determine whether changes in defense mechanisms predict decreased symptoms and examine the temporality between changes in defense mechanism and depressive symptoms, and furthermore examine if these changes are specific to PDT.

There is meager empirical evidence for the role of these theoretical construct as mediators and more research is needed to determine if such variables actually mediate the effects of psychotherapy, and to what degree they are specific to CBT or PDT [23, 41, 53]. Furthermore, our understanding of MDD may improve from mediator research. Focusing on one helpful aspect in therapy that is followed by improvement in symptoms may shed light on important aspects of the disorder [48].

## Causal inference in randomized groups

Methods in causal inference have rapidly developed over the last decades, and are now widespread in most fields, both in social science and medicine. They are also useful to assess mechanisms and effect heterogeneity (variation across individuals) between treatment and outcome. The relevant causal concepts are mediation and interaction, in which the counterfactual-based approach has become standard, in line with the book of VanderWeele [81]. A counterfactual-based approach involves “potential outcomes” to conceptualize causation, by conceiving of what might have occurred had the treatment been otherwise than it was. If the outcome would have been different due to a different treatment, then it is meant that the treatment “causes” (affects) the outcome. Although it is difficult to draw causal conclusions for individuals based on a dataset, one can make inference about such effects on average for a population under specific assumptions. A necessary assumption in mediation is temporality. Repeated measures allows for ensuring that the treatment precedes the mediator, which again precedes the outcome.

Methods for mediation can help to understand mechanisms and pathways through intermediates in a causal effect of treatment on an outcome. The motivation to assess mediation can be interest in refinement in a treatment intervention. This might be done by improving components in the treatment that target a particular mechanism. Before proceeding with such a refinement, it is desirable to know the extent to which the mechanism targeted is an important pathway from treatment to outcome. Empirical studies of mediation may help to assess relative importance of various mechanisms.

Effect heterogeneity is often explained by an additive interaction, or moderation (prescriptive factor), when different subgroups of individuals defined by one variable somehow alters the treatment effect, the moderator being the variable that defines the subgroups. Additive interactions can help to determine which subgroups would benefit most from a treatment method or even if it is harmful for some. Assessing interaction can also shed light on mechanisms themselves [81].

Explanations of a treatment effect, by mediation and interaction are not unrelated. The phenomena can simultaneously be present, and will be if the portion of the effect that operates through a particular mechanism is different from the effect of intervening on that mechanism, so that treatment both affects and interacts with the mediator.

Assumptions that are needed to assess mediation, are in general much stronger than those needed to assess an overall causal effect of treatment. With randomized treatment group assignment, the causal group difference (overall causal effect) is automatically identifiable, in contrast to the mediated effect. With this background we designed the Mechanisms Of change in Psychotherapy (MOP) study.

## Methods/design

### Overview

The Mechanisms of Change in Psychotherapy (MOP) study is a randomized controlled trial (RCT) where patients diagnosed with MDD are allocated to either CBT or PDT. It is a clinical trial designed to measure the effects of the two treatments, attempting to answer the questions of what works for whom and how. Clinical assessments are conducted at baseline, several times during therapy, at the end of therapy, and at follow-up investigations 1 and 3 years after treatment termination. All treatment sessions are videotaped. The outcome measures comprise a large range of clinical variables enabling studies on process analyses. Potential moderators and mediators from CBT and PDT will be tested in both treatment arms. A qualitative study will be nested within the RCT.

### Aims

Primary outcome of the study will be depressive symptoms assessed with both Hamilton Depression Rating Scale (HDRS) and Beck Depression Inventory II (BDI II). As we expect PDT and CBT to be equally effective, the overall aim of the MOP study is to examine mechanisms of change in psychotherapy and examine specific markers of psychotherapy outcome (moderators). Consequently, the planned analysis refers to the putative moderators and mediators. The study will focus on research questions within the following areas:

#### Moderators of outcome

1. Are there certain patient characteristics that moderate the outcome of CBT and/or PDT?
  - If so, which patient characteristic differentially influence outcome in the two treatment conditions?
  - Could clusters of moderators [Personalized Advantage Index (PAI)] be identified and predict treatment outcomes for subgroups of patients?

Bases on previous studies, we hypothesize that patients with more avoidant personality traits, a more distancing attachment style, more paranoid symptoms/traits, and higher baseline anxiety levels report better outcomes of CBT than PDT.

Among relevant patient characteristics to be examined as potential moderators are: socio-demographic variables (e.g. gender, age, ethnicity), severity of symptoms (e.g. depression severity, comorbidity), rumination, functional impairment, childhood trauma and neglect, attachment style, personality functioning, interpersonal problems, reflective functioning, and previous psychotherapy.

#### Mediators of change

1. Does improvement occur through different or similar change processes (mechanisms) in the two treatment modalities?

We will test the following theoretically based hypotheses:

- In CBT symptom and functional improvement will be mediated by changes in negative automatic thoughts, dysfunctional attitudes, attributional styles, underlying cognitive schemas, and the learning/development of compensatory skills.
- In PDT symptom and functional improvement will be mediated by improved self-understanding/insight and emotional awareness, less harsh self-view, more adaptive personality functioning, more mature defense mechanisms, and increased reflective functioning.

#### Common versus specific factors

Do common factors (e.g. therapeutic alliance, therapist competence and adherence to the therapy model) differentially influence outcome in the two treatment modalities?

We will test the following hypothesis:

- Therapeutic alliance is rated significant higher (WAI) for patients who have improved (lower BDI II scores) in the PDT arm compared to patients who have improved in the CBT arm.
- No significant differences in the patients score regarding therapist competence and adherence to the therapy model (WAI) will be obtained between the two groups for patients who improves (BDI II).

### Ethics

The study will be conducted in accordance with the ethical guidelines as described in the Helsinki—declaration (Declaration of Helsinki, 2013). The Regional Committee for Medical and Health Research Ethics has approved the study protocol (REK: 2016/340). Patients will receive both written and oral information about the study, before they are asked to give their written consent to participate. All data will be stored according to approval. Both of the two treatment modalities offered are well-established psychotherapy methods in which patients have responded, on average, equally well. The treatment are manualized [11, 32]. The therapists are experienced and specifically trained, and the therapies will be supervised throughout the study period. Patients who do not wish to participate in the study will receive treatment as usual at Nydalen and Vinderen outpatient clinics, Oslo, Norway. The patients are informed that they can withdraw from the study whenever they want to, and that this will have no consequences for their further treatment at the outpatient clinic.

### User involvement

A service user with relevant diagnosis and experience of therapy have been recruited to participate in the formation of the qualitative study interview and give feedback on the general structure, implementation and conduction of the study from a patient participants point of view.

### Eligibility criteria

#### Inclusion criteria

Inclusion criteria are currently fulfilling the criteria of a MDD according to the DSM-IV (based on a clinical interview and MINI), age 18–65 years, the ability to understand, write and speak a Scandinavian language, and willingness and ability to give informed consent.

#### Exclusion criteria

Exclusion criteria are current or past neurological illness, traumatic brain injury, current alcohol and/or substance dependency disorders, psychotic disorders, bipolar disorder type 1, developmental disorders, and mental retardation. Patients are not excluded for other comorbid psychiatric conditions in order to capture a representative sample of depressed individuals.

### Patients

**Fig. 1:**
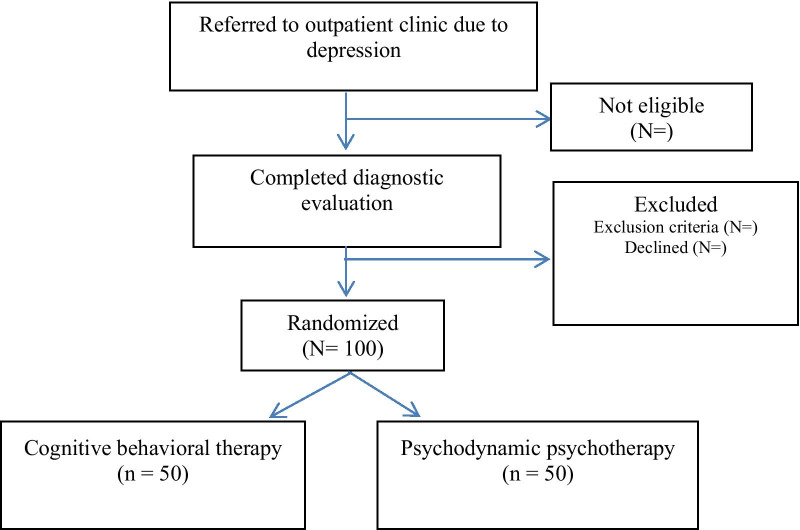
Flow diagram describing the MOP study: randomized controlled trial comparing cognitive behavioral therapy and psychodynamic psychotherapy

Patients are recruited among patients referred with depressive symptoms to two outpatient clinics in Oslo, South Eastern health region, Norway (Nydalen DPS, and Vinderen DPS). Recruitment commenced at Nydalen Outpatient Clinic, Oslo University Hospital (OUS) in January 2017, while Vinderen psychiatric outpatient unit, Diakonhjemmet hospital startet recruiting patients June 2018. In total one hundred patients will be recruited Fig. 1.

### Randomization procedures and methods used to minimize bias

#### Recruitment and baseline procedures

The patients are first screened for inclusion and exclusion criteria. Patients eligible for the study are given oral and written information about the project and are invited to a second interview after 2–3 days. Where written consent is obtained, baseline assessment commences.

When these assessments have been completed a trial ID is assigned and patients are allocated to one of the two therapies. To minimize bias, the design is single blind, i.e. outcome assessors at pre and post treatment and further follow-up evaluations will not be aware of assigned study condition. If blindness is broken, all subsequent assessment will be carried out by an alternative evaluator [36].

### Assessments and outcomes

The baseline and follow up clinical examination will be conducted by a group of experienced clinicians who will complete a training and reliability program at the Division of Psychiatric Treatment Research, Oslo University Hospital. In addition, the raters will receive supervision on a regular basis by experienced raters and clinician. Consensus meetings will be held in order to assure reliability of assessments.

### Pre-treatment

#### Interviews

Before randomization each patient is diagnosed according to the DSM-IV criteria using the M.I.N.I. [54] and the SCID II [34]. The Psychodynamic Interview (PI) modified after Malan and Sifneos [60, 73] is used to assess psychological functioning through the Psychodynamic functioning scale (PFS) [46]. The PFS consists of 6 scales that explore Quality of family relationships, Quality of friendships, Quality of romantic/sexual relationships, insight, tolerance for affect and problem solving and adaptive capacity. The psychodynamic interview also taps quality of object relations (QOR-2 relations to others and relation to family). The Depression Specific Reflective Functioning interview (DSRF) [71] is used to assess reflective functioning, and the Global Assessment of Psychosocial Functioning (GAF) addresses level of functioning and symptom severity [70]. Hamilton Depression Rating Scale (HDRS) [39] assesses level and characteristics of depression.

#### Self-report

An extensive battery of self-report questionnaires is used; The Working Alliance Inventory (WAI-SR_1) [43] assess alliance, the Experience in Close Relationships (ECR) [20] evaluates attachment style and Inventory of interpersonal problems (IIP-64) [2] taps interpersonal distress. The Structural Analysis of Social Behaviour (SASB) [37] also assess interpersonal and intrapsychic interactions, and Severity Indices of Personality Problems-Short Form (SIPP-SF) [82] is used to assess personality problems.

Questionnaires that assess childhood trauma, Childhood Trauma Questionnaire (CTQ) [17], work and social adjustment, WSAS [65], emotional awareness/alexithymia, the Toronto Alexithymia scale (TAS-20) [4] will also be used at several time points during therapy and at follow up.

Metacognitions are assessed using Meta Cognitive Questionnaire (MCQ-30) [85], cognitive insight using Becks Cognitive Insight Scale (BCIS) (Aaron T. [13], dysfunctional attitudes using the Dysfuntional attitude scale (DAS) [14] Rumination related scales are used, including the Positive Beliefs about Rumination Scale (PBRS) [69] Negative Beliefs about Rumination Scale (NBRS) and Ruminative Response Scale (RRS) [78].

Depressive symptoms are assessed using Becks Depression Inventory (BDI) [15] and manic/hypomanic symptoms using Hypomania Checklist-32 [3]. Levels of anxiety are assessed using GAD-7 [74]. In addition sociodemographic are recorded and health related quality of life is assessed by Short-Form 12 (SF-12) [84].

### During therapy

During therapy the patients are assessed with self-report questionnaires at weeks 3, 8, and 16. At 3 weeks, the WAI-SR_1 is used.

At 8 weeks, the WAI-SR_1, SIPP-SF, WSAS, GAD-7, TAS-20, BDI, PBRS, NBRS, SAS, ECRI, MCQ-30, RRS, BCIS and DAS are used.

At 16 weeks, WAI-SR_1, SIPP-SF, TAS-20, BDI, PBRS, NBRS, WSAS, GAD-7, SASB, ECR, RRS, MCQ-30, BCIS and DAS are used.

### Post treatment

At the end of treatment patients are interviewed and assessed by the same team of evaluators as in the pre-treatment assessment. The patients are interviewed using the M.I.N.I. to ascertain if they still satisfy the diagnostic criteria for a current depressive episode. The posttreatment assessment also includes the Psychodynamic Interview (PI), The Depression Specific Reflective Functioning interview (DSRF), Global Assessment of psychosocial Functioning (GAF) and Hamilton Depression Rating Scale (HDRS).

Most of the self-report questionnaires from the baseline assessments are used, including the WSAS, SF-12, BDI, SIPP-SF, TAS-20, PBRS, NBRS, SAS, ECR, MCQ-30, RRS, BCIS, GAD-7, DAS, IIP-64 and HCL-32. In addition sociodemographic variables are recorded.

### Follow up assessments

#### 1 year follow up

The M.I.N.I interview will be used to ascertain if the patient still satisfy the diagnostic criteria for a current depressive episode. The 1 year follow up also includes the Psychodynamic Interview (PI), The Depression Specific Reflective Functioning interview (DSRF), Global Assessment of psychosocial Functioning (GAF) and the Hamilton Depression Rating Scale (HDRS).

Most of the self-report questionnaires from the baseline assessments are used, including the WSAS, SF-12, BDI, SIPP-SF, TAS-20, PBRS, NBRS, SAS, ECR, MCQ-30, RRS, BCIS, GAD-7, DAS, IIP-64 and HCL-32. In addition sociodemographic variables are recorded.

#### 3 year follow up

The MINI interview will be used to ascertain if the patient still satisfy the diagnostic criteria for a current depressive episode. The 3 year follow up also includes the Psychodynamic Interview (PI), The Depression Specific Reflective Functioning interview (DSRF), Global Assessment of psychosocial Functioning (GAF) and the Hamilton Depression Rating Scale (HDRS).

Most of the self-report questionnaires from the baseline assessments are used, including the WSAS, SF-12, BDI, SIPP-SF, TAS-20, PBRS, NBRS, SAS, ECR, MCQ-30, RRS, BCIS, GAD-7, DAS, IIP-64 and HCL-32. In addition, sociodemographic variables are recorded.

## Intervention: CBT compared to PDT for treatment of patients with MDE

### Therapists

The psychotherapy with CBT and PDT are conducted by therapists from Nydalen and Vinderen psychiatric outpatient clinic, who have completed a minimum of two-year training in CBT and PDT. In addition, all therapists receive specific training in the CBT and PDT study manuals prior to commencing as therapists in the study. All treatment sessions are videotaped and independent psychiatrists will carry out assessment of treatment fidelity on a selection of random tapes.

### Treatments

Patients are randomized to 16 weekly sessions of CBT followed by 3 monthly booster sessions, or 28 weekly sessions of PDT. This approach was chosen in an attempt to design the study as close to clinical practice as possible (ecological validity). In designing the study, we discussed this issue with both experienced clinicians and psychotherapy researchers. They seemed to agree that it was no firm clinical or empirical evidence to have more than 19 sessions of CBT and too little with less than 28 sessions of PDT. However, both arms have the same measurements point (during therapy, at end of therapy (28 weeks) and at the 1 and 3 years follow-up). This approach is in line with other studies comparing CBT and PDT [35]. All sessions last 45 min.

#### Psychodynamic psychotherapy (PDT)

Short-term psychodynamic psychotherapy (STPP) is a well-known treatment mode. The dynamic therapy in MOP is based on the general psychodynamic principles described by Gabbard [32]. The STPP manual used in the First Experimental Study of Transference–Interpretations [45] describes the specific features for time-limited therapy used in the trial. A case formulation is made in collaboration with the patient during the first sessions, describing (1) symptoms and problems, (2) precipitating stressors or events, (3) predisposing life events or stressors, (4) mechanisms that links preceding categories together, and finally 5) what to focus in the therapy The time-limited approach is further specified in guidelines for patient-information at the start of treatment. The dynamic treatment is mainly exploratory in nature. Patients are encouraged to explore sensitive topics and repetitive interpersonal patterns, including exploration of the patient-therapist relationship [79]. Exploration and interpretation of transference, i.e. linking interpersonal patterns to transactions between the patient and the therapist will be used with moderate intensity.

#### Cognitive behavioural therapy (CBT)

Cognitive therapy for depression by Beck et al. [11] will be used as treatment manual. The CBT manual describes the goal directed framework of the intervention. The first sessions will focus on the therapeutic alliance and engagement of the patients to therapy. Psychoeducation will be provided. The intervention will focus on both short and long term goals. Different cognitive techniques, described in the manual, will be applied to reach these goals. The last part of therapy will focus on consolidation and relapse prevention.

### Statistical analysis plan

An causal graph (DAG) of mediation is shown in Fig. 1, with randomized treatment $A$ that corresponds to the two treatment groups in MOP, mediator $M$, outcome $Y$ that corresponds to symptoms of depression, and mediator-outcome confounders $C$. The longitudinal nature of the data in MOP, is well suited to assess mediation, in that group assignment can, with certainty temporally precede the mediator which again one can be sure of temporally precedes the outcome, to assure causal effects. Repeated assessments, both on the mediator and symptoms of depression, can be used to generalize the simple setting with one assessment of each. The basic concepts are easiest illustrated in the simple setting. Traditional methods to study mediation have important limitations concerning interactions and non-linearities [9], in contrast to the counterfactual-based approach, which is able to decompose a total effect of treatment on the outcome into indirect effect (through the mediator), and direct effect (direct relative to the mediator), even in the presence of an interaction between treatment and mediator. It can be argued that the interaction term should be included, even if it’s not statistically significant. In order to specify assumptions and identifiability of effects, the following definitions of indirect / direct effects are needed. In general, let ${a}^{*}$ and $a$ be t**w**o levels of treatment, here the two groups of CBT or PDT.

$CDE\left(m\right)$: The controlled direct effect expresses how much symptoms of depression would change on average if the mediator where fixed at level $m$ uniformly in the whole population, but the treatment group where changed from ${a}^{*}=0$ to $a=1$

$NDE$: The natural direct effect expresses how much symptoms of depression would change if the treatment were set at level $a=1$ versus level ${a}^{*}=0$, but for each individual the mediator were kept at the level it would have taken, for that individual, if she had been in the reference group (${a}^{*}=0$) (effect of treatment group assignment on symptoms of depression, that would remain if the pathway from treatment to mediator were disabled).

$NIE$: The natural indirect effect expresses how much symptoms of depression would change on average if the treatment group were fixed at $a=1$, but the mediator were changed from the level it would take if ${a}^{*}=0$ to the level it would take if $a=1$ (effect of treatment group assignment on symptoms of depression that operates by changing the mediator).

**Fig. 2:**
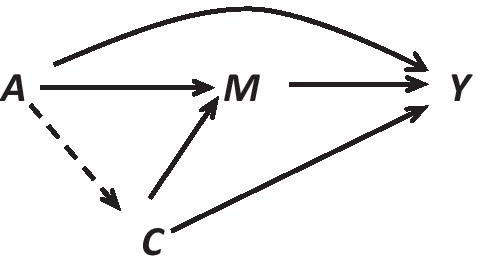
Causal diagram for mediation, A: treatment, M: mediator, Y: outcome and C: measured confounders

Simple regression models will suffice to estimate the direct and indirect effects above, but some confounding control assumptions are needed. Randomization of treatment group guarantees unbiased estimation of total effect of treatment group on symptoms of depression, but not unbiased estimation of direct / indirect effects [81]. In order to be able to estimate the $CDE\left(m\right)$, an assumption of no unmeasured confounding for the mediator—depression relationship is needed (all confounders are measured and controlled for). This is also a necessary assumption to be able to estimate $\mathrm{NDE}$ and $\mathrm{NIE}$. In addition, no mediator- depression confounder should be affected by treatment group assignment (the dashed arrow in Fig. 2 has to be absent). If such an influence (arrow) is plausible, the natural direct and indirect effects are not identifiable. The controlled direct effect can still be identified with a structural mean model (SMM), and estimated by g-estimation (Vansteelandt 2016).

Randomization of treatment group also strengthens assessment of effect heterogeneity/moderation, from other variables than the mediator. This is estimated by including an interaction term in the regression.

Direct and indirect effects are generalizable to time-varying mediators, but with similar assumptions as mentioned above. Without time-varying confounders for the mediator—depression relationship that are affected by the group-assignment, the natural direct/indirect effects are identifiable (in addition to the controlled direct effect) in longitudinal equivalents. With a time-varying confounder affected by group-assignment, only the longitudinal equivalent of the controlled direct effect is identifiable, and can be estimated with the marginal structural model [81]. Also, a time-varying moderator effect is identifiable in a structural nested mean model, and can be estimated by g-estimation (Vansteelandt 2016).

## Personalized Advantage Index (PAI)

Multivariate regression and machine learning approaches, in line with the study by van Bronswijk et al. [80]*,* will be used to compute the PAI.

### Power analysis

With reference to a t-test comparison of two independent groups (of equal size) as the total effect, a sample size (each group) of 26 / 64 would provide 80% power (significance level of 5%) to detect a difference of a large (0.8) / medium (0.5) effect size, respectively. With respect to power and sample-size calculations for mediation analysis, current literature is somewhat limited [81]. Kenny and Judd [51] showed that in many settings, power to detect indirect effects is higher than that for total effects, and power to detect direct effects is less than that for total effects. In the present study, this should imply a reasonable power for direct effects, to a less extent indirect effects and even less for moderators. However, the Personalized Advantage Index (PAI) including a group of moderators should alleviate this problem for identifying interactions.

### Nested qualitative study

A qualitative study will be nested within the quantitative RCT study.

There is a scarcity of qualitative studies exploring patient’s experiences of both CBT and PDT. To our knowledge, only two studies have explored how patients experience different aspects of PDT and CBT respectively [66]. The main aim of this subproject will be to expand and add nuances to the experiences patients suffering from MDD after PDT and CBT treatment. This may further broaden our understanding of the processes in psychotherapy and help clinicians’ better tailor the treatment for each individual patient. Approximately 10 patients who have received CBT and 10 patients who have received PDT will be asked to participate in the qualitative study. A strategic sampling will be established based on sex and age. A semi-structured interview guide is developed with the help of a user service group. The interview lasts for 45 to 60 min, and is tape-recorded and transcribed. A phenomenological/hermeneutic approach will be used to analyse the data.

The interview covers the following themes: Experiences with PDT and CBT respectively, experiences of processes that were followed by improvement, experiences of aspects that were unhelpful, and experiences with external factors (family, friends, work, school etc.) that were important for improvement or lack of improvement. The themes will be explored in details to detect the mechanisms leading to the reported changes that take place during the course of treatment (identifying theory of change).

## Discussion

Major depressive disorder (MDD) is a prevalent psychiatric condition associated with significant disability, mortality and economic burden. While CBT and PDT are found to be equally effective for the treatment of patients with depression, little is known about which patient characteristics (moderators) may be associated with differential outcomes of CBT and PDT, and through what kind of therapeutic processes and mechanisms (mediators) improvements occur in each therapy mode. There are presently only theoretical assumptions, sparsely supported by research findings, about what moderates and mediates the treatment effects of CBT and PDT. Given the lack of findings in this field of research, the National Institute for Health and Clinical Excellence (Depression, 2009) called for the examination of moderators of response to CBT and PDT in the treatment of moderate and severe depressive disorders as a research recommendation in order to improve patient care.

The use of moderators and mediators to predict of what treatment would best suit a given individual has come to be referred to as *personalized medicine,* and discovering these factors is considered one of the major challenges in health care research today [38]. The lack of knowledge of what treatment is most beneficial for an individual patient often leads to valuable time being wasted as patients are not immediately receiving the treatment that could most benefit them.

The Mechanisms Of change in Psychotherapy (MOP) study aims to address a most pressing question in current psychotherapy research; what helps for whom. A representative and heterogeneous out-patient sample of patients suffering from depression with comorbidities such as anxiety and PDs is included from the pool of patients admitted to two outpatient clinics in Oslo, Norway. The two groups will be compared on a number of variables to investigate potential moderators and mediators of clinical and functional improvement. All patients will be closely examined prior to, during and after treatment, all sessions will be video-taped and a qualitative study will focus on harvesting information of patient´s experience of therapy and experienced mechanisms of change.

The study´s sound methodological structure address many of the shortcomings of previous psychotherapy studies, and also aim to fruitfully engage practitioners of different theoretical backgrounds in a collaborative endeavor with researchers. Better understanding of the therapeutic process may increase our ability to refine our treatments, to make them more effective, and to personalize them for the specific needs of the individual patient.

### Trial status

Protocol version 2 (November 2018). The first patient was included February, 2017. The study is ongoing and has recruited 63 patients (February, 2020). The last patients will be assessed three years after end of treatment in December 2023 (Table 1).

**Table 1:** Overview: assessments administered at research baseline and each follow-up point throughout the trial

Assessment points	Interviews	Self-report questionnaires	Therapist questionnaires
Baseline (0 weeks)	M.I.N.I., SCID-II, PFS, DSRF, GAF, Hamilton	SF-12, WSAS, CTQ, BDI, SIPP-SF, TAS-20, PBRS/NBRS, SASB, ECR, MCQ-30, RRS, GAD-7, BCIS, DAS, IIP-64, HCL-32	WAI, FWC-58, Therapist adherence
After session 3		WAI	WAI, FWC-58
After session 8		SF-12, WSAS, WAI, BDI, SIPP-SF, TAS-20, PBRS/NBRS, SASB, ECR, MCQ-30, Rumination scale, GAD-7 BCIS, DAS	WAI, FWC-58
After session 16		SF-12, WSAS, WAI, BDI, SIPP-SF, TAS-20, PBRS/NBRS, SASB, ECR, MCQ-30, Rumination scale, GAD-7, BCIS, DAS,	WAI, FWC-58
After 28 weeks/post treatment	M.I.N.I. (depression module only) PFS, DSRF, GAF, Hamilton	SF-12, WSAS, WAI, BDI, SIPP-SF, TAS-20, PBRS/NBRS, SASB, ECR, MCQ-30, Rumination scale, GAD-7, BCIS, DAS, IIP-64, HCL-32	WAI, FWC-58
1 year follow up	M.I.N.I.(depression module only) PFS, DSRF, GAF, Hamilton	SF-12, WSAS, BDI, SIPP-SF, TAS-20, PBRS/NBRS, SASB, ECR, MCQ-30, Rumination scale, GAD-7 BCIS, DAS, IIP-64, HCL-32	
3 year follow up	M.I.N.I. (depression module only), GAF, Hamilton	SF-12, WSAS, BDI, SIPP-SF, TAS-20, PBRS/NBRS, SASB, ECR, MCQ-30, Rumination scale, GAD-7 BCIS, DAS, IIP-64, HCL-32	

WAI-SR_1, Working Alliance Inventory; WSAS, The Work and Social Adjustment Scale; SF-12, The 12-item Short Form Health Survey; CTQ, Childhood Trauma Questionnaire; BDI, Beck Depression Inventory; SIPP-SF, Severity Indices of Personality Problems-Short Form; TAS-20, Toronto Alexithymia Scale; PBRS, Positive Beliefs about Rumination Scale; NBRS, Negative Beliefs about Rumination Scale; SASB, Structural Analysis of Social Behaviour; ECR, Experiences in Close Relationships Inventory; MCQ-30, The Metacognitions Questionnaire 30; RRS, Ruminative Responses Scale; BCIS, Beck Cognitive Insight Scale; GAD-7, Patient Health Questionnaire; DAS, Dysfunctional Attitude Scale; IIP-64, Inventory of interpersonal problems; HCL-32, Hypomania Checklist-32

## Data Availability

The datasets used and/or analysed during the current study are available from the corresponding author on reasonable request.

## Abbreviations

BDI: Becks Depression Inventory
CBT: Cognitive Behaviour therapy
DSM-V: Diagnostic and Statistical Manual of Mental Disorders—version IV
GAF: Global Assessment of Functioning
M.I.N.I.6.0.0.: Mini International Neuropsychiatric Interview
MDD: Major Depressive Disorder
MDE: Major Depressive Episode
MOP: Mechanisms Of change in Psychotherapy
PI: Psychodynamic Interview
PSI: Psychodynamic Functioning Scale
QOR-2: Quality of object relationship Score
RCT: Randomized Controlled Trial
SCID-II: Structured Clinical Interview for DSM-IV-(Personality disorders)
STPP: Short Term Psychodynamic/psychoanalytic psychotherapy
WAI: Working Alliance Inventory
